# Draft Genome Sequence of Streptomyces aureoverticillatus HN6, a Strain Antagonistic against Fusarium oxysporum f. sp. *cubense* Race 4

**DOI:** 10.1128/MRA.00210-20

**Published:** 2020-06-04

**Authors:** Rong Di, Yee Chen Low, Lanying Wang, Yanping Luo

**Affiliations:** a Department of Plant Biology, Rutgers, The State University of New Jersey, New Brunswick, New Jersey, USA; b College of Plant Protection, Hainan University, Hainan, China; Broad Institute

## Abstract

Streptomyces aureoverticillatus HN6 was isolated in Hainan, China. It is highly inhibitory to Fusarium oxysporum f. sp. *cubense* race 4 (FOC4), which causes banana *Fusarium* wilt. The HN6 genome was fully sequenced and assembled. Bionformatic analysis shows that the HN6 genome contains at least 208 genes involved in antibiotic biosynthesis.

## ANNOUNCEMENT

Fusarium oxysporum f. sp. *cubense* race 4 (FOC4), the causal agent of the banana *Fusarium* wilt disease, poses a serious threat to banana production worldwide (1, 2). In an effort to search for biocontrol microorganisms for the management of banana *Fusarium* wilt disease, we have isolated an actinomycete from the soil on the tropical Hainan Island, China (3). Our previous findings have shown that this actinomycete isolate, HN6, is highly inhibitory to the growth of FOC4, and its methanol extract has broad-spectrum antifungal activities (3). We have identified HN6 as a strain of Streptomyces aureoverticillatus by morphological, biochemical, physiological, and cultural characterizations and 16S rRNA gene sequencing (3).

HN6 was grown to log phase in Gause’s number 1 synthetic medium, and its genomic DNA was isolated using the cetyl trimethylammonium bromide (CTAB) method (3). The HN6 genomic DNA was sent to the Beijing Genomic Institute (BGI) (Shenzhen, China). The library, containing 500-bp inserts, was constructed with a TruSeq DNA kit and sequenced by BGI using an Illumina HiSeq 2000 instrument to generate 90 paired base reads. The raw reads were trimmed using Quality Trimmer 1.1.5 (4) and Cutadapt 1.16.6 (5) of the Galaxy platform. Low-quality ends were trimmed with the parameters of a minimum quality score of 20 and window size of 3. The 3′ adapter was also removed, with the parameter of a minimum overlap length of 5. A total of 150,608 cleaned reads were generated, representing an average sequence depth of 99.99. The cleaned reads were assembled using the Velvet assembler 1.2.10.1 (6) with an optimized k-mer resulting in 4 contigs. The total size of the assembly is 9,738,784 bp with a G+C content of 71.51%. The assembled sequence was analyzed using Glimmer 3.02 (7). The analysis showed that HN6 has 8,248 genes with a total length of 8,534,217 bp, averaging 1,035 bp per gene, with 87.63% gene-to-genome coverage. Tandem Repeats Finder 4.04 (8) was used to find 2,486 repetitive sequences with a total of 117,608 bp. There are also 2,194 minisatellites and 46 microsatellites. In addition, 18 rRNAs and 64 tRNAs were identified using RNAmmer 1.2 (9) and tRNAscan-SE 1.23 (10), respectively. Default settings were applied for all software used in the sequence analysis. Gene functions were predicted using the BLAST tool by aligning the assembled sequences to the Gene Ontology (GO) (11), KEGG (12), SWISS-PROT (13), GenBank nonredundant (NR) (14), and COG (15) databases. The COG functional classification showed that HN6 has 366 genes involved in secondary metabolite biosynthesis, transport, and catabolism, among which at least 208 genes are involved in the biosynthesis of antibiotics, including penicillin, streptomycin, and tetracycline. Additionally, at least 36 genes were identified to be involved in antibiotic resistance based on an analysis using the Antibiotic Resistance Genes Database (ARDB) (16) and the Comprehensive Antibiotic Research Database (CARD) (17).

These genome sequence data suggest HN6’s strong potential for secondary metabolism and provide valuable information for the discovery of bioactive compounds and their implications in its antifungal activities. Gene expression analysis and bioassays are needed to dissect the production of the bioactive compounds by HN6 in order to fully utilize this isolate as a biocontrol agent for plant disease management.

### Data availability.

The whole-genome sequence of HN6 has been deposited in GenBank under the accession numbers CP048641 and CP048642 for the assembled sequences and the accession number SRR11584258 for the trimmed raw reads. The BioProject number is PRJNA605209, and the BioSample number is SAMN14052186.
